# Multi-disciplinary management of type 1 and 2 skin tears using a silver-based hydrofiber dressing

**DOI:** 10.1097/MD.0000000000035112

**Published:** 2023-09-15

**Authors:** Shu-Ping Chou, Ya-Hui Yen, Ya-Ting Tseng, Chiou-Ping Chen, Hsin-Hua Ke, Yi-Kung Lee, Yung-Cheng Su, Honda Hsu

**Affiliations:** a Department of Nursing, Dalin Tzu Chi Hospital, Buddhist Tzu Chi Medical Foundation, Dalin, Taiwan; b Department of Nursing, Taichung Tzu Chi Hospital, Buddhist Tzu Chi Medical Foundation, Taichung, Taiwan; c Department of Emergency Medicine, Dalin Tzu Chi Hospital, Buddhist Tzu Chi Medical Foundation, Dalin, Taiwan; d School of Medicine, Tzu Chi University, Hualien, Taiwan; e Department of Emergency Medicine, Ditmanson Medical Foundation Chia-Yi Christian Hospital, Chiayi City, Taiwan; f Division of Plastic Surgery, Dalin Tzu Chi Hospital, Buddhist Tzu Chi Medical Foundation, Dalin, Taiwan; g School of Medicine, Institute of Medical Sciences, Tzu Chi University, Hualien, Taiwan.

**Keywords:** hydrofiber, silver, skin tear

## Abstract

Skin tear is a common problem encountered in the emergency department. If it is not properly managed, it can lead to wound infection, skin necrosis and a need for further surgical intervention and skin grafting. Current management is to cleanse the wound, replace the thin skin tear followed by coverage with a dressing that is inducive for wound healing. Several dressings have been suggested for the coverage of these wounds. But, up to now, there has been no mention of the use of a silver-based hydrofiber dressing in the management of this condition. The objective of this study was to explore the use of a silver-based hydrofiber dressing for the management of paper-thin skin tears. We retrospectively reviewed all patients with Type 1 or 2 skin tears that had undergone management using a silver-based hydrofiber dressing between October 2019 and October 2020. Demographic data and medical history was obtained by retrospective chart review. Data that was collected included: age, sex, comorbid illnesses, defect location, defect size, complications, number of times the silver-based hydrofiber dressing was replaced and the number of days required to achieve complete wound healing. A total of 65 patients were included in the study. There were 42 males and 23 females. There were 28 patients whose age was greater then 85 years old, of which 14 patients were over 90 years old. The mean number of outpatient visits was 2. The mean defect size was 33 cm^2^ (range 1 cm × 1 cm to 18 × 10 cm). The mean number of days required for total wound healing was 13 days (range 7–21). We did not encounter any patients that required further surgical debridement or split-thickness skin grafting. The use of a silver-based hydrofiber dressing was well tolerated by the elderly population as it provided an easy, efficient, economical and effective form of management of skin tears. We suggest that a silver-based hydrofiber dressing can be used as a first-line treatment method for type 1 and 2 skin tears.

## 1. Introduction

Skin tear is a common problem encountered in the Emergency Department, but is often not high on the list of priorities for management by Emergency Department staff due to more serious life and death situations faced every day. However, if this condition is not properly managed, it can lead to wound infection, skin necrosis and a need for further surgical intervention and skin grafting. It is a problem that affects patients across all age spectrums but is especially prevalent in the elderly and in chronically ill individuals. In the elderly population, minor traumas such as wheelchair or chair injuries, transferring from bed to chair, minor falls, bumps, and even tape removal can result in skin tears.

The prevalence of skin tears is estimated to be between 1.1% and 41.2%. The highest prevalence is seen in long-term care facilities.^[1]^ Payne and Martin classification of skin tears was used originally to describe the severity of skin tears. Category I skin tears were defined as skin tears without any tissue loss, Category II skin tears were defined as tears associated with partial tissue loss, and Category III tears were defined as tears associated with complete tissue loss.^[2]^ This classification system was first revised by the skin tear audit research classification system and has now been updated by the International Skin Tear Advisory Panel (ISTAP) Classification System. Skin tears according to ISTAP can now be classified into type 1 (no skin loss), Type 2 (partial flap loss) or Type 3 (total flap loss).^[3]^

The current recommended management is for a complete assessment of the wound to determine the type of skin tear. The wound should be thoroughly cleaned. Wound healing will only take place once surface debris, necrotic tissue, biofilms, and foreign body have been removed. The thin skin tear should be replaced, realigned and if possible re-approximated.^[4]^ Hydrogel, alginate, lipido-colloid based mesh and foam dressings, soft silicone, foam, calcium alginate dressings, absorbent clear acrylic dressings, and skin glue have all been described as dressings that can be used for the coverage of these wounds.^[5,6]^

But to date there has been no mention of using a silver-based hydrofiber dressing (Aquacel Ag, Convatec, London, UK) in the management of this common condition. The objective of this study was to explore the use of a silver-based hydrofiber dressing for the management of type 1 and 2 skin tears. We assessed the time required to achieve complete wound healing as well as whether additional wound treatment therapies were required.

## 2. Methods

We conducted a single-center, retrospective, noncomparative study to evaluate the efficacy of using a silver-based hydrofiber dressing in the management of skin tears. All patients who between October 2019 and October 2020 had presented to the Emergency Department with type 1 or 2 skin tear and had undergone skin tear management using a silver-based hydrofiber dressing (Aquacel Ag, Convatec, London, UK) were included in the study. Patients with deep, infected, heavily contaminated and actively bleeding wounds that did not respond to application of local pressure were excluded. type 3 skin tears as well as avulsed full thickness flaps containing subcutaneous tissue were also excluded.

If skin tear management was performed with any other method they were excluded from the study. Demographic data that was collected included: age, sex, comorbid illnesses, defect location, defect size, complications, the number of times the silver-based hydrofiber dressing was replaced and the number of days required to achieve complete wound healing. (Table 1). The study protocol was approved by the institution review board of our hospital.

**Table 1:** Summary of patient demographics and results.

Total no of patients	65
Male	39
Female	26
Mean age (in yr)	83
Location of skin tears	
Upper extremity	28
Lower extremity	25
Mixed areas	5
Face	2
Chest	1
Mean defect size (cm^2^)	33 (Range 1–180)
No of days till total wound healing	13 (Range 7–21)
Wound infection (no of patients)	3

No = number.

## 3. Interventions

**Figure 1.**
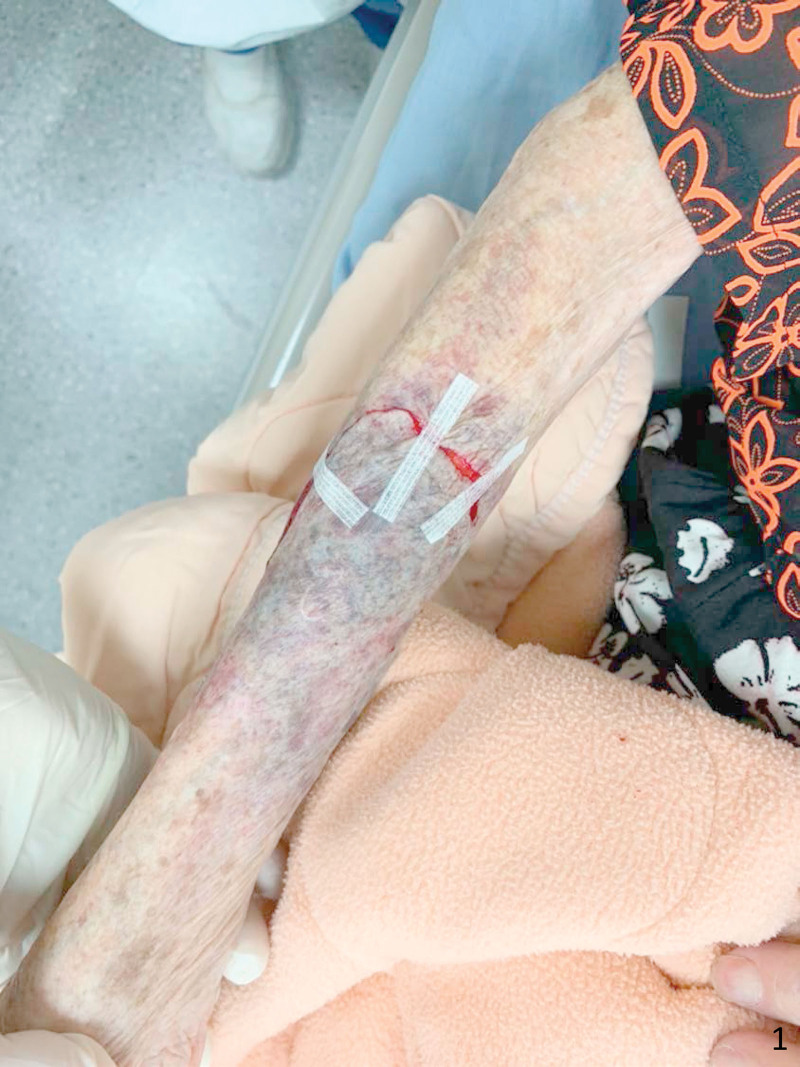
The skin tear was realigned as much as possible. Occasionally, remanent defect can be seen.

**Figure 2.**
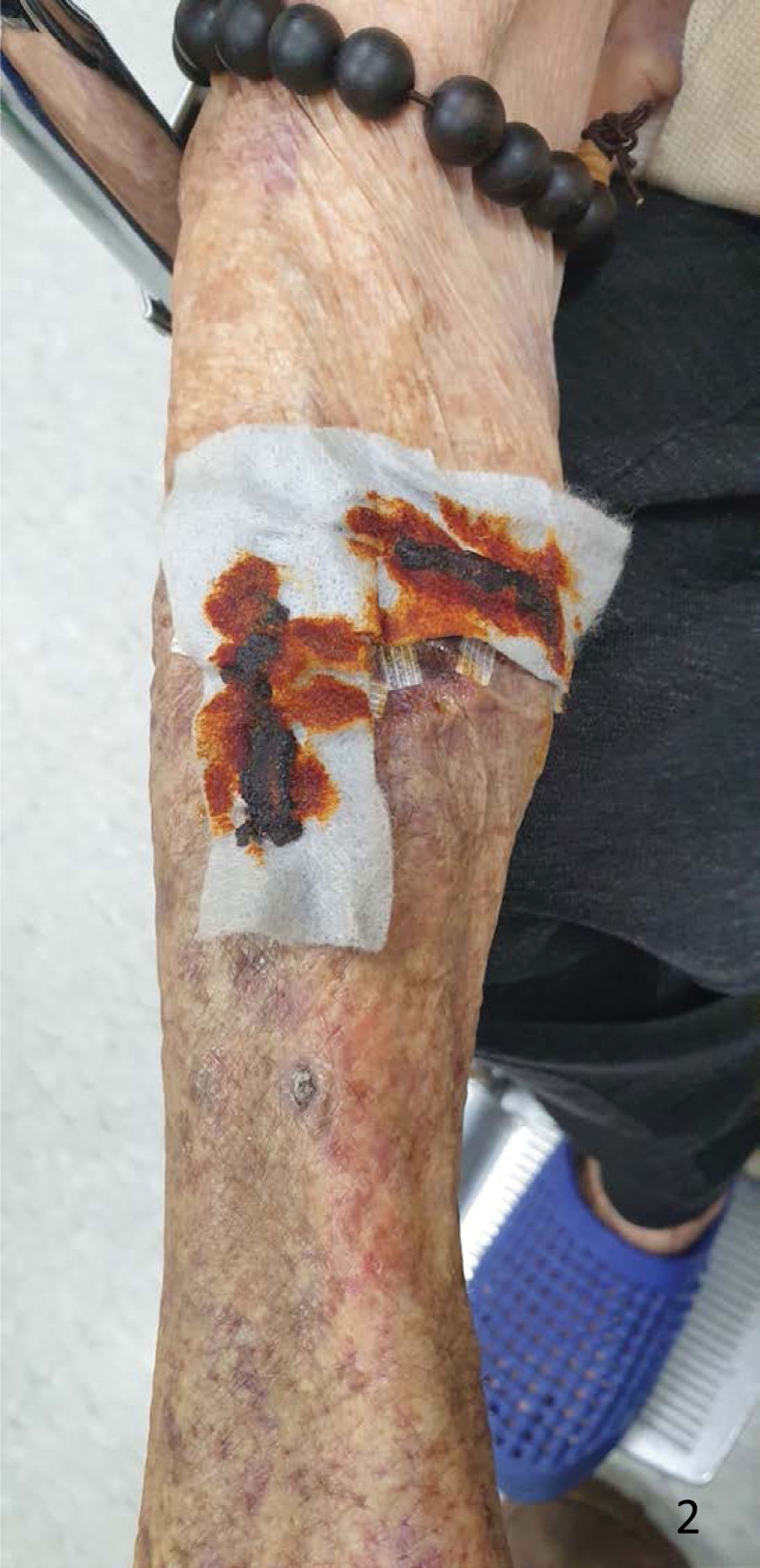
The silver-based hydrofiber dressing was examined at the outpatient clinic 3 days later to ensure that it remained dry.

**Figure 3.**
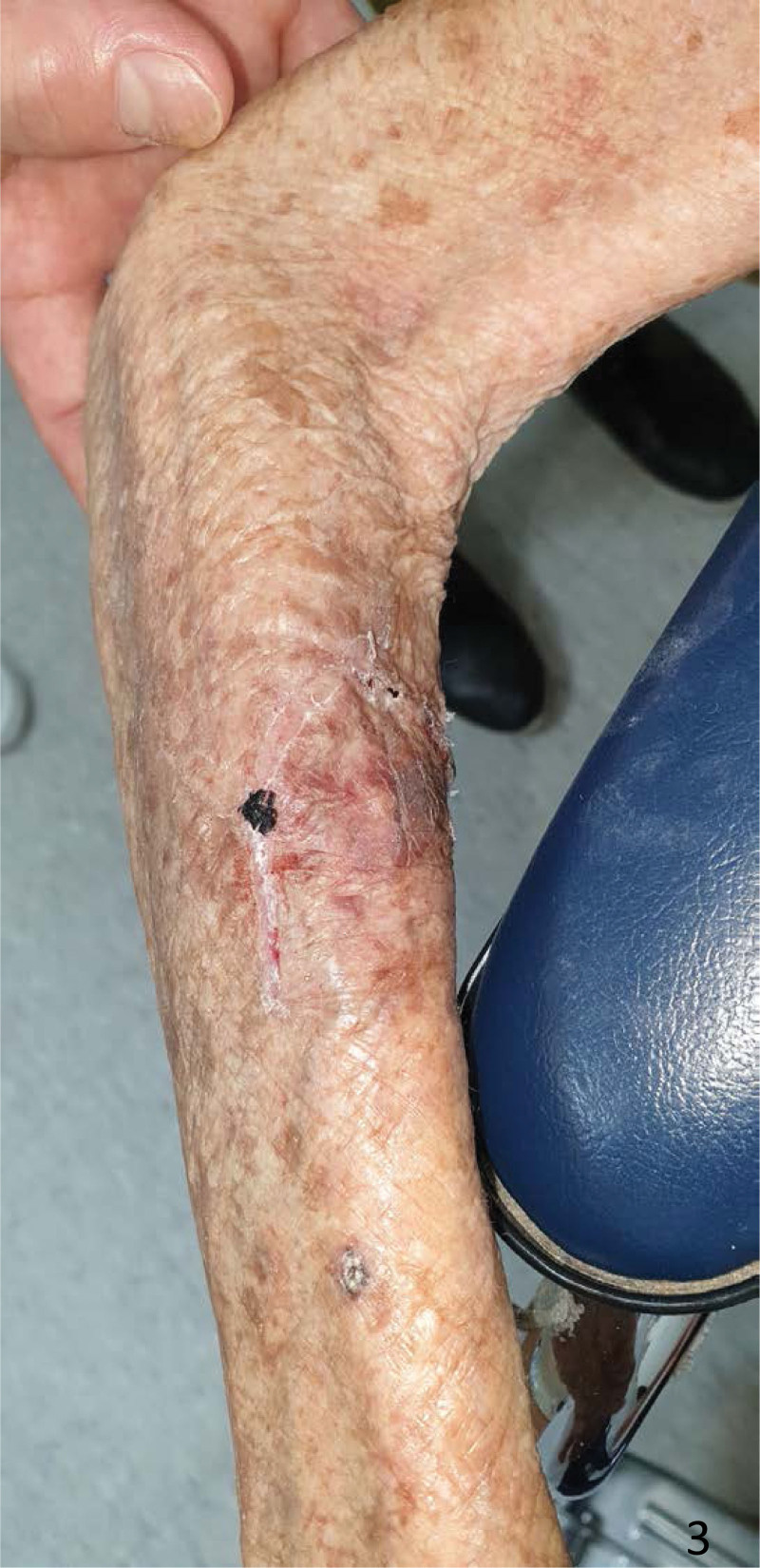
Removal of the silver-based hydrofiber dressing showing complete wound healing.

On presentation at the emergency department, all wounds were cleansed with normal saline and bleeding was stopped with the application of direct local pressure. In patients with type 1 and 2 skin tears, the avulsed skin tears were placed back, realigned and their edges were re-approximated as much as possible. Surgical adhesive tape (Steri-Strips; 3M, St. Paul, MN) was used to assist in fixation after realignment. At times, complete re-approximation was not possible and remanent raw surfaces could be seen (Fig. 1). The silver-based hydrofiber dressing was placed over the re-approximated skin tear as well as over the raw surface. Gauze was placed over this dressing and compression bandage was applied to ensure that the hydrofiber comes into direct contact with the skin tear and the raw surface. Sutures and staples were not used in any of these patients. The patients and their families were instructed on how to remove the gauze dressing and to inspect and assess the silver-based hydrofiber dressing the following day. If silver-based hydrofiber dressing was dry, new gauze was placed over this. They were to do this on a daily basis. The patients were seen again 3 days later in the plastic and reconstructive surgery outpatient clinic (Fig. 2). Repeat assessment of the dressing was made by the physician in the outpatient clinic and if it was dry, the patient and their family were encouraged to continue changing the gauze dressing daily. A follow-up appointment was made for 1 week later, at which time the silver-based hydrofiber dressing was removed and the percentage of wound healing was determined. If the silver-based hydrofiber dressing was still firmly attached then removal was not undertaken and a further appointment was made for another week later. The need for silver-based hydrofiber dressing was stopped only when complete wound healing was observed (Fig. 3). The steri-strips often fell off at this time or were easy to remove once complete healing of the skin tear was seen. There was a variation in the timing of the removal of the silver-based hydrofiber dressing as this had to be adjusted to the days that the patients could return to the outpatient department for follow-up. In all of these patients, no further procedures were required. If the silver-based hydrofiber dressing looked saturated, it was removed and replaced with a new silver-based hydrofiber dressing. If there was infection of the wound, the silver-based hydrofiber dressing was removed and was changed to Povidone-Iodine coated dressing (Meidine, JenSheng, Taiwan), which was applied daily. The patient was placed on an oral antibiotic at this time.

## 4. Results

**Figure 4.**
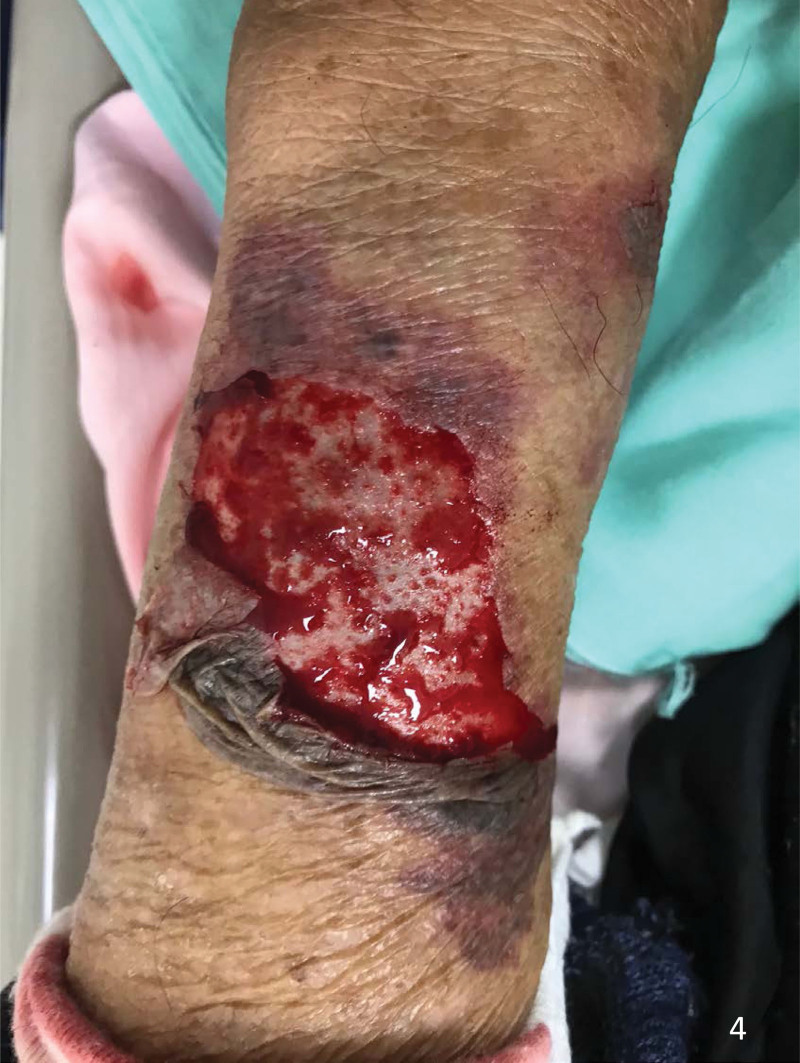
An 86-year-old male with skin tear of the right forearm due to minor trauma when he fell from his chair. A type 2 skin tear was seen.

**Figure 5.**
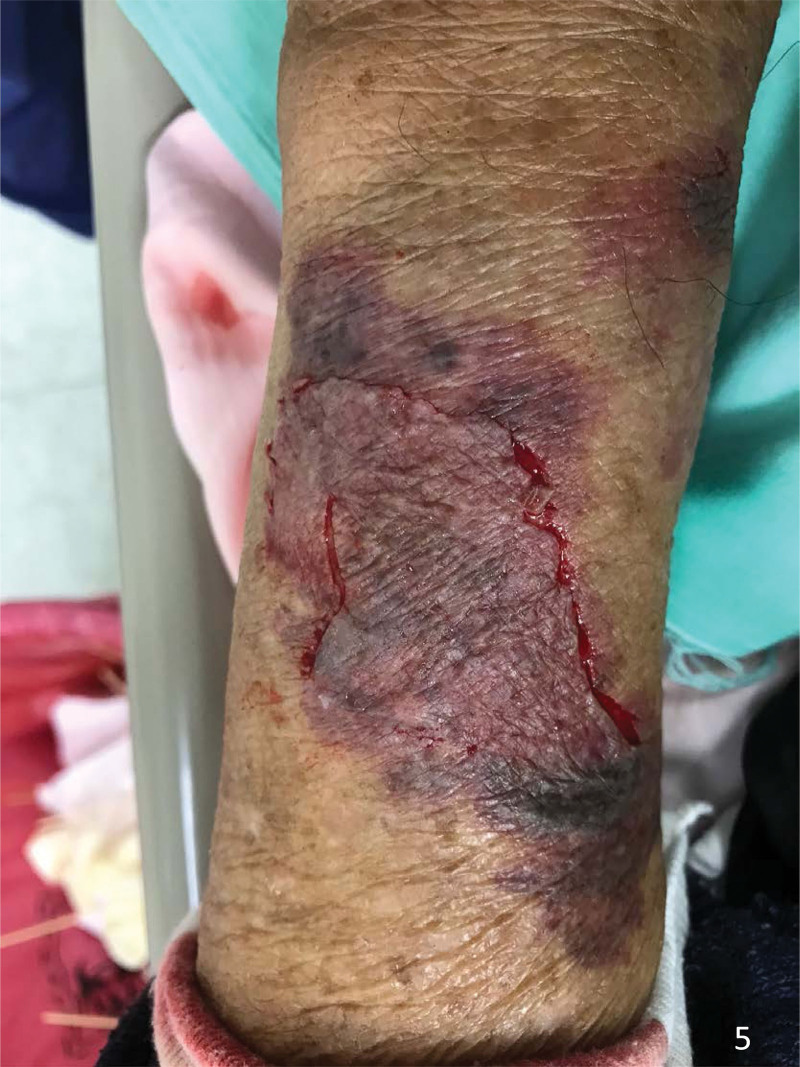
The skin tear was replaced back, realigned and covered with a silver-based hydrofiber dressing.

**Figure 6.**
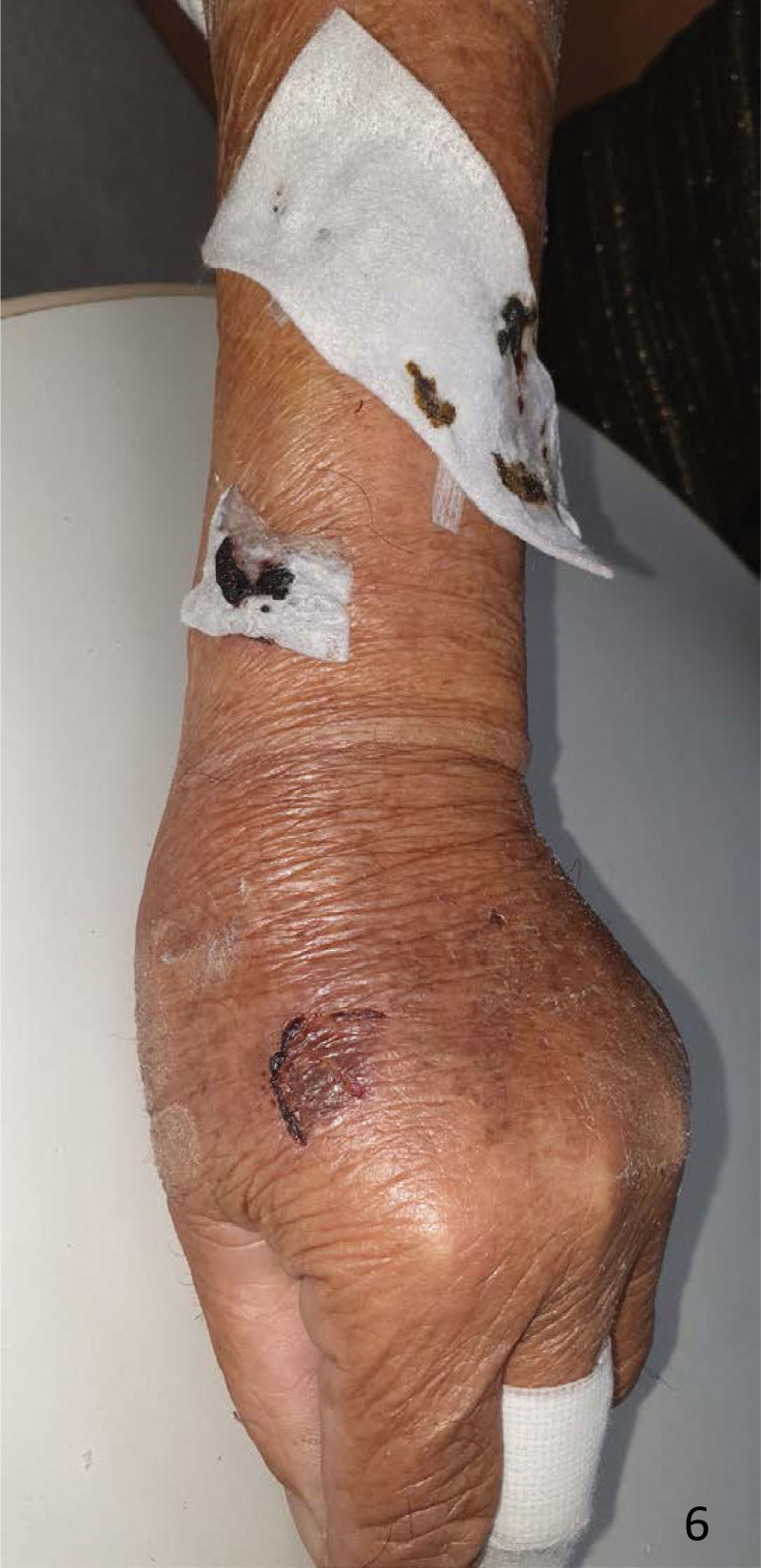
Review of the dressing 3 days later showed it to be dry.

**Figure 7.**
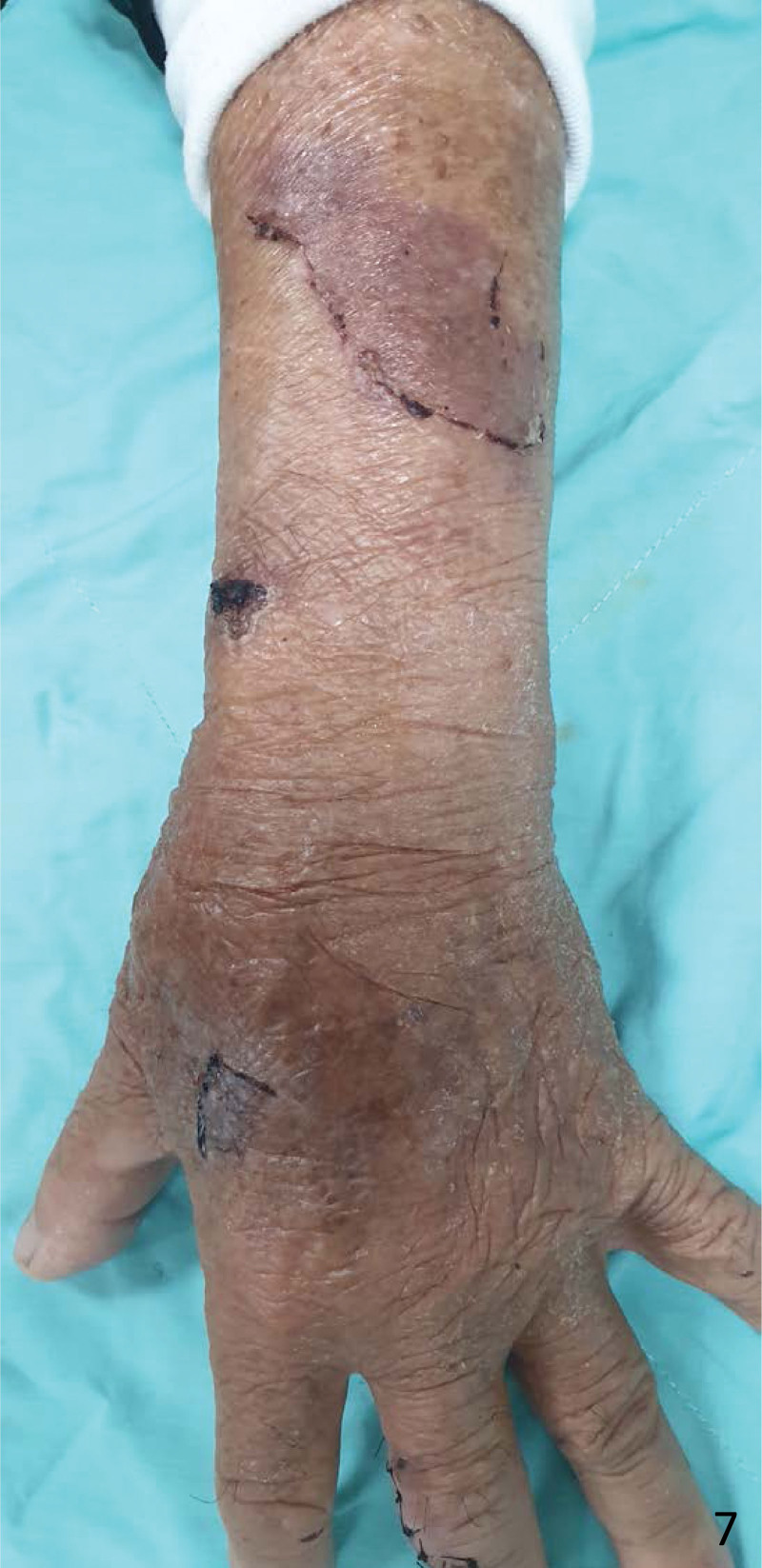
The dressing was removed 17 days later and showed complete healing of the skin tear.

**Figure 8.**
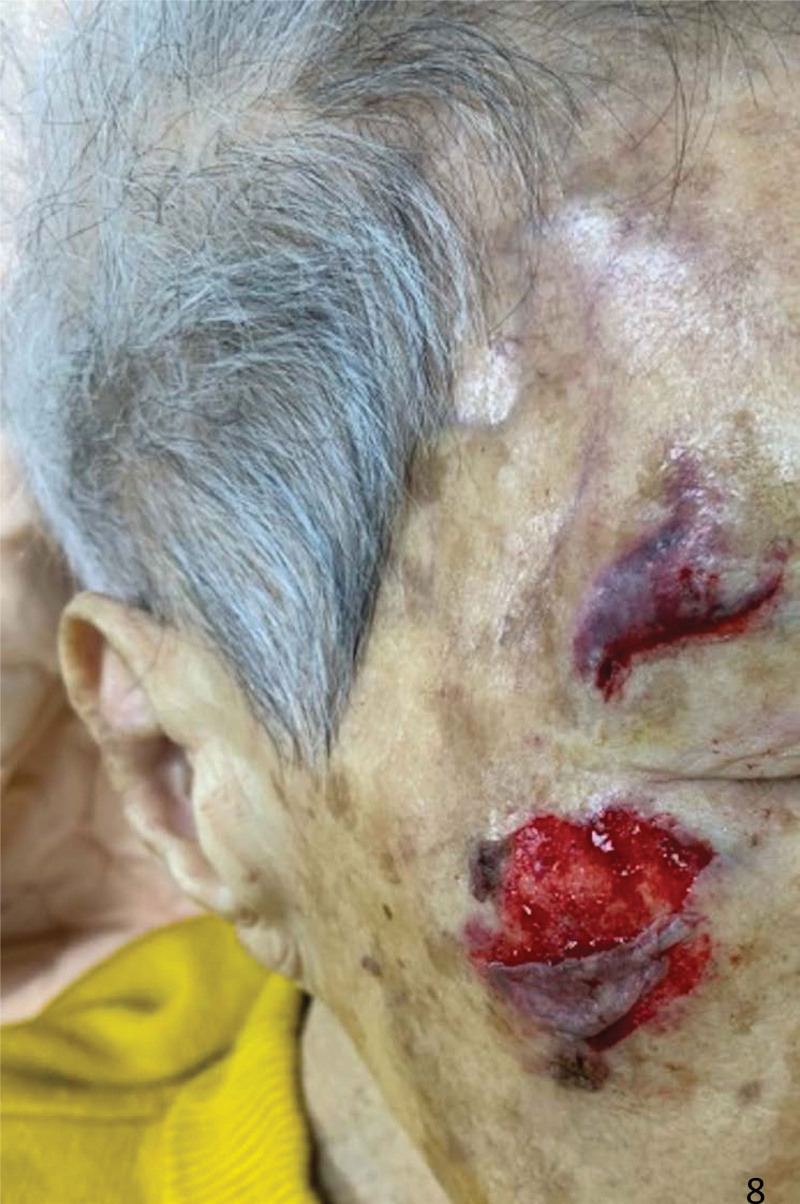
A 82-year-old woman who fell and sustained type 2 skin tears to her right face.

**Figure 9.**
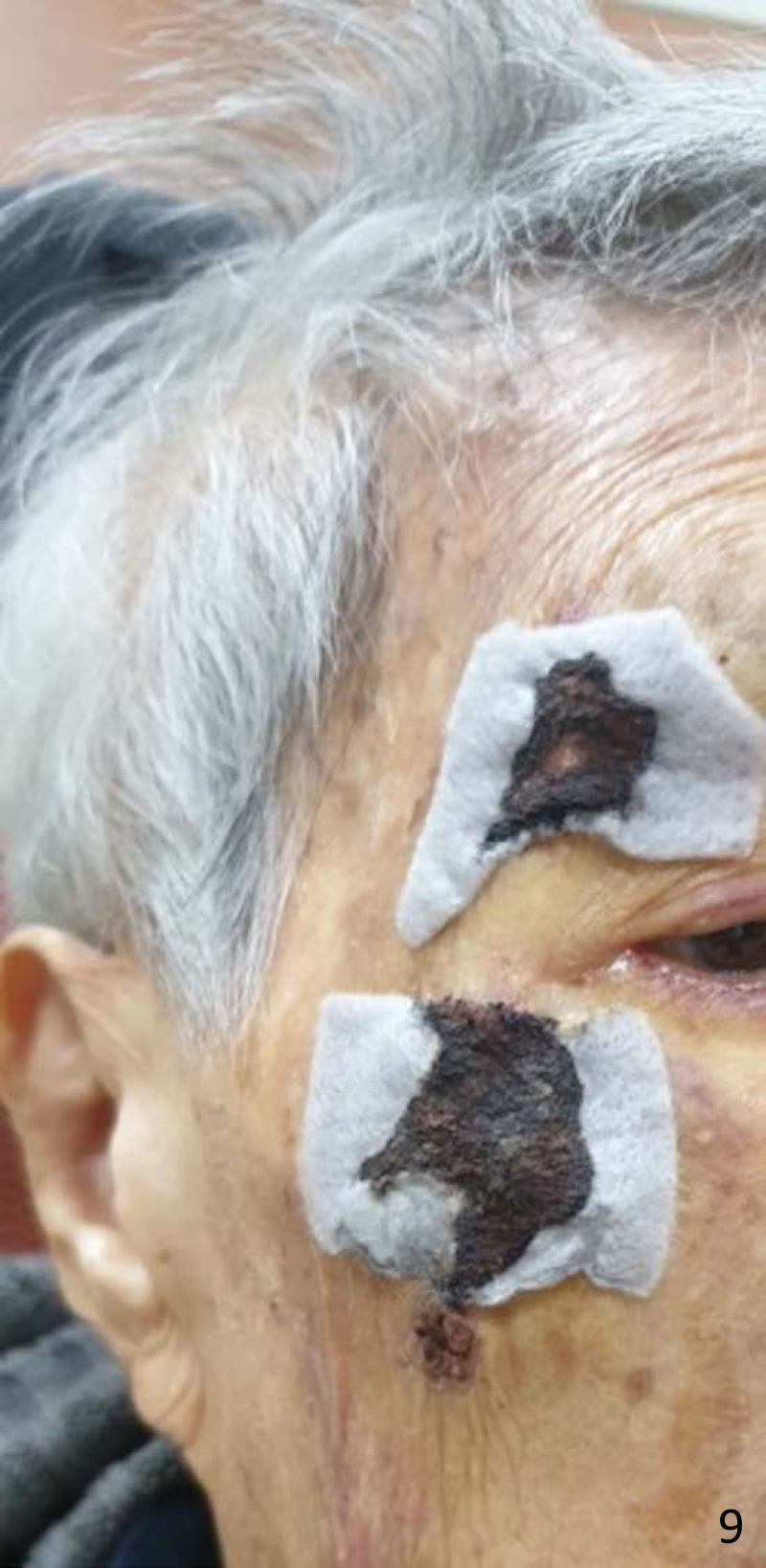
Silver-based hydrofiber dressing was used for wound coverage after the skin tear was realigned.

**Figure 10.**
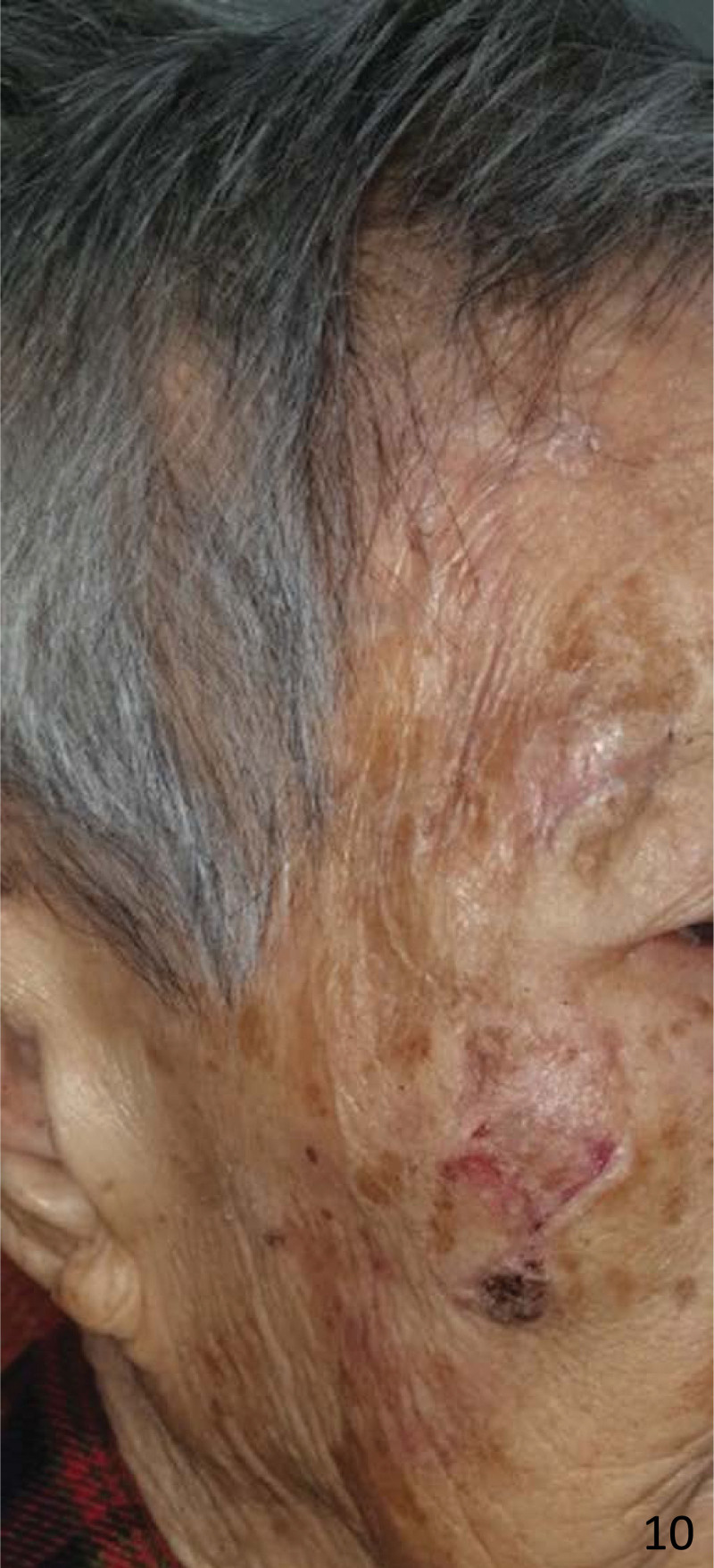
The dressing was removed 10 days later and showed complete healing of the skin tear.

A total of 73 patients underwent management of their skin tears using a silver-based hydrofiber dressing but 8 patients were lost during follow-up. A final total of 65 patients was included in the study. There were 42 males and 23 females. Their mean age was 83 years old. Interestingly, there were 28 patients in the study whose age was >85 years old, of whom 14 patients were over 90 years old. There were 28 patients whose skin tears were located in the arms, forearms and hands, 25 patients whose skin tears were located in the thighs, pretibial area and over the dorsal feet. In 5 patients the skin tear covered a mixed area of both the upper and lower extremity, in 2 patients the skin tears were over the face, and there was 1 patient where the skin tear was located over the anterior chest. There were no patients who required further surgical interventions or were admitted during follow-up. The mean number of days required for total wound healing was 13 days (range 7–21). The mean number of outpatient visits was 2. This did not include the initial visit to the Emergency Department. There were 3 patients who developed an infection of the wound. In these patients, the silver-based hydrofiber dressing was removed and changed to a Povidone-Iodine coated gauze dressing. They were instructed to change the dressing daily and were placed on oral antibiotics. Daily wound care was performed until the wound healed by secondary intention. In these 3 patients, it took an average of 24 days before there was complete wound healing. We were fortunate not to encounter any patients who required further surgical debridement and split-thickness skin grafting. The mean defect size in these patients was 33 cm^2^ (range 1 cm × 1 cm to 18 × 10 cm) (Table 1). Two illustrative cases are shown in Figures 4–10

## 5. Discussion

This study showed that even though skin tears are common, it is often mis-managed. This is in part due to the incorrect diagnosis of a skin tear and the inability to distinguish it from other skin lesions.^[7]^ The lack of a standardized, universally accepted classification system may have contributed significantly to these variations.^[8]^ Despite their significant impact, skin tears are often unrecognized and underreported, resulting in delayed or inappropriate treatment.^[9,10]^ The term “skin tear” is not commonly used, and is often referred to as a “laceration” or “abrasion.”^[8–11]^ A skin tear, however, is very different from a laceration which is defined as a cut or tear of soft body tissue^[12]^ where soft tissue includes muscle, fatty and fibrous tissue, tendons, ligaments, nerves, and blood vessels. A skin tear does not include any of these.^[10]^

Skin tears were defined by Payne and Martin as “a traumatic injury occurring on the extremities of older adults as a result of shearing or friction forces, which separate the epidermis from the dermis.”^[13]^ This was later revised in 1993 to “a traumatic injury occurring principally on the extremities of older adults as a result of shearing or friction forces which separate the epidermis from the dermis (partial-thickness wound) or which separate both the epidermis and the dermis from the underlying structures (full-thickness wound).”^[2]^ The definition has since been revised further by the ISTAP. It now defines skin tears as ‘traumatic wounds caused by mechanical forces, including removal of adhesives. Severity may vary by depth (not extending through the subcutaneous layer).^[8]^ The ISTAP Classification System classifies skin tears into 3 types: Type 1 (no skin loss), Type 2 (partial flap loss) or Type 3 (total flap loss).^[3]^ But the term “flap” used in this classification may be misleading, as a flap by definition is a type of tissue elevated from the body which has its own intact blood supply in comparison to a graft which does not have its own blood supply and relies on growth of new blood vessels. As such skin tears which do not have their own blood supply should not be called flaps as this will lead to a confusion in the terminology. We would suggest using an anatomical description of the 3 types of skin tears, that is, type 1 (no dermal epidermal loss), type 2 (partial dermal epidermal loss), and type 3 (total dermal epidermal loss). But regardless of the definition, skin tears occur commonly in individuals of extreme age, in the critically ill or medically compromised, and in those requiring assistance with daily activities. At present, dressings commonly used for the management of skin tears include hydrogel, alginate, lipido-colloid based mesh and foam dressings, soft silicone, foam, calcium alginate dressings, absorbent clear acrylic dressings, and skin glue.^[5,6]^ But there has been no description of the use of a silver-based hydrofiber dressing for the management of this commonly seen problem.

This retrospective, noncomparative study shows that a close working relationship was required between the emergency department personnel who performed the initial management and the plastic and reconstructive surgeons who undertook the follow-up management of the patients. The use of a silver-based hydrofiber dressing was shown to have functioned well in the management of type 1 and 2 skin tears within the Emergency Department setting and is well accepted by the elderly and the general population. The application was easy and painless. The daily task of caring for the dressing and wound was stress-free and well tolerated by family members looking after the patients or even by the patients themselves. Out of all the patients, there were only 3 cases where management was unsuccessful as a result of infection. The patients in this study were in the very large majority of cases extremely elderly, with an average age of 83 years old. There were 28 patients who were older than 85 years old and 14 patients who were over 90 years old. In the case of extreme age, it is important to ensure that wounds can be taken care of easily and without fuss. When treating elderly patients one should further take into account that degenerative osteoarthritis or rheumatoid arthritis may be present in the hands, leading to deterioration of fine motor skills. They are more susceptible to failing eyesight, as well as failing memory. They might often be living alone or in some circumstances it is 1 elderly family member looking after the other (elderly wife looking after elderly husband or vice versa). This category of patient will not adopt or tolerate procedures required to care for the wound if these become complicated. In the example of this study, all that was required of patients at home was to change to gauze covering the silver-based hydrofiber dressing and to ensure that the dressing did not get wet.

Silver has been used widely in wound management for many years to help control local infection. Historically, silver can be dispensed as metallic (silver foil), solution (e.g., silver nitrate), or cream (e.g., silver sulfadiazine). Ionic silver (Ag^+^) has received renewed interest as a prophylactic antimicrobial agent in wound dressings due to its broad spectrum antibacterial range. There are various mechanisms that can be used to explain the antimicrobial effect of ionic silver. Silver interferes with the cytochromes of microbacteria and additionally also interferes with components of the microbial electron transport system, binds DNA, and inhibits DNA replication. There is at present little evidence of emerging microbial resistance to silver.^[14–18]^

Aquacel Hydrofiber dressing (ConvaTec, London, UK) is a moisture- retention dressing. It consists of soft nonwoven sodium carboxymethylcellulose fibers. This becomes gel-like when it comes in contact with wound fluid. This gel promotes a moist wound healing environment while at the same time it retains wound exudates by vertical absorption. Fibrin that collects between the hydrofiber and the wound surface acts as an adhesive, fixing the dressing in place, allowing the adherence of the dressing to the wound.^[19–25]^

It is based on these 2 properties that we used a silver-based hydrofiber for fixation of the paper-thin skin tears. Hydrofiber dressing has been applied to many types of wound care with favorable results and cost-effectiveness.^[19–25]^ Silver-based hydrofiber has been used to treat partial thickness burns both in the adult and pediatric population. It has been used in chronic wounds, nonischemic diabetic foot ulcers, split-thickness skin graft donor sites and in acute traumatic wounds.^[26–32]^ Even though the International Wound Infection Institute does not recommend the use of antimicrobial dressings in noninfected wounds, silver-based dressings and povidone-iodine dressings have been used successfully in the management of surgical and traumatic wounds.^[29]^ We chose a silver-based dressing in the management of skin tears as we felt that the wounds were often caused due to trauma and not in a sterile environment such as in the operating theater. The wound could be contaminated at the time of the injury despite the subsequent best efforts of the Emergency Department to cleanse the wound. On occasion these patients did not present immediately to the Emergency Department but only some hours later when discovered by their families. In these cases we were worried about wound infection and the formation of biofilms. It is for these reasons that we chose a silver-based hydrofiber instead of a plain hydrofiber.

A further benefit of this method was that it avoided the need for sutures or staples. Even though sutures and staples are not advised in these types of wounds, often patients still mistakenly undergo primary repair. By using this dressing, it avoids the risk of needle stick injuries and decreases the treatment time required for the management of these types of injuries. This is very important in the Emergency Department where time is always severely limited. In this study, we managed the skin tears based on a similar concept to the management of skin grafts as thin skin tears are basically skin grafts. We used steri-strips in some of the cases to fixate the re-approximated skin tear. Although this is not in line with current ISTAP recommended best practice, it is a common method practiced in Plastic and Reconstructive surgery for skin graft fixation.^[33–36]^ The steri-strips are left in place and they often detach by themselves once complete healing of the skin tear occurs at around 2 to 3 weeks. By this time the steri-strips have lost their adhesive properties and fall off by themselves or can be removed atruamatically. We do not recommend the removal of steri-strips prior to this as it can induce further trauma to the thin, fragile skin.

This method was also welcomed by the patients themselves as they avoid the pain associated with the injection of local anesthetics and suturing. The method was indeed so simple that, following complete healing of the wound, some of the patients themselves asked to purchase additional silver-based hydrofiber dressings to keep at home should further similar injuries occur in the future.

## 6. Limitations

The limitations applicable to this study was that it was carried out in a single-center and was a retrospective, noncomparative study. There were also 8 patients that were lost during follow-up. It is feasible that these patients presented instead to another hospital with wound infections. A further limitation is that no plain hydrofibers were used in this study and so we were not able to ascertain if the use of plain hydrofibers could have similar results.

## 7. Conclusion

This study showed that the use of a silver-based hydrofiber dressing was well tolerated by the elderly population as it provided an easy, efficient, economical, and effective form of management of skin tears. We suggest that a silver-based hydrofiber dressing can be used as a first-line treatment method for type 1 and 2 skin tears.

## Acknowledgments

We would like to thank Martin Laing, a great friend, for reviewing the English.

## Author contributions

**Conceptualization:** Honda Hsu.

**Data curation:** Shu-Ping Chou, Ya-Hui Yen, Ya-Ting Tseng, Chiou-Ping Chen, Hsing-Hua Ke, Honda Hsu.

**Formal analysis:** Honda Hsu.

**Investigation:** Shu-Ping Chou, Ya-Hui Yen, Ya-Ting Tseng, Chiou-Ping Chen, Hsing-Hua Ke, Honda Hsu.

**Methodology:** Yung-Cheng Su, Honda Hsu.

**Project administration:** Yi-Kung Lee, Yung-Cheng Su, Honda Hsu.

**Resources:** Yung-Cheng Su.

**Software:** Yung-Cheng Su.

**Supervision:** Yi-Kung Lee, Yung-Cheng Su.

**Validation:** Shu-Ping Chou, Ya-Hui Yen, Ya-Ting Tseng, Chiou-Ping Chen, Hsing-Hua Ke, Honda Hsu.

**Visualization:** Honda Hsu.

**Writing – review & editing:** Shu-Ping Chou, Ya-Hui Yen, Ya-Ting Tseng, Chiou-Ping Chen, Hsing-Hua Ke, Yi-Kung Lee, Yung-Cheng Su, Honda Hsu.

**Writing – original draft:** Honda Hsu.

## References

[1] Van Tiggelen H Beeckman D. Skin tears anno 2022: an update on definition, epidemiology, classification, aetiology, prevention and treatment.

[2] Payne RL Martin ML. Defining and classifying skin tears: need for a common language. Ostomy Wound Manage. 1993;39:16–20, 22. 8397703

[3] LeBlanc K Baranoski S Holloway S. Validation of a new classification system for skin tears. Adv Skin Wound Care. 2013a;26:263–5. 23685526 10.1097/01.ASW.0000430393.04763.c7

[4] Sibbald G Orstead H Coutts P. Best practice recommendations for preparing the wound bed: update 2006. Wound Care Canada. 2006;4:19–29. 10.1097/01.ASW.0000280200.65883.fd 17620740

[5] LeBlanc K Baranoski S. Skin tears: state of the science: consensus statements for the prevention, prediction, assessment, and treatment of skin tears. Adv Skin Wound Care. 2011;24:2–15. 21876389 10.1097/01.ASW.0000405316.99011.95

[6] Xu X Lau K Taira BR. The current management of skin tears. Am J Emerg Med. 2009;27:729–33. 19751631 10.1016/j.ajem.2008.05.015

[7] LeBlanc K Alam T Langemo D. Clinical challenges of differentiating skin tears from pressure ulcers. EWMA J. 2016;16:17–23.

[8] LeBlanc K Campbell K Beeckman D. Best practice recommendations for the prevention and management of skin tears in aged skin. Wounds Int 2018; Available at: https://www.woundsinternational.com/resources/details/istap-best-practice-recommendations-prevention-and-management-skin-tears-aged-skin. 10.1097/WON.0000000000000481 30395131

[9] LeBlanc K Baranoski S. Skin tears: finally recognized. Adv Skin Wound Care. 2017;30:62–3. 28106632 10.1097/01.ASW.0000511435.99585.0d

[10] Idensohn P Beeckman D Campbell KE. Skin tears: a case-based and practical overview of prevention, assessment and management. J Community Nurs. 2019a;33:32–41.

[11] Rayner R Carville K Leslie G. A review of patient and skin characteristics associated with skin tears. J Wound Care. 2015;24:406–14. 26349021 10.12968/jowc.2015.24.9.406

[12] National Library of Medicine. Laceration versus puncture wound. 2019. Available at: https:// medlineplus.gov/ency/imagepages/19616.htm.

[13] Payne RL Martin ML. The epidemiology and management of skin tears in older adults. Ostomy Wound Manage. 1990;26:26–37. 2306325

[14] Burd A Kwok CH Hung SC. A comparative study of the cyto- toxicity of silver-based dressings in monolayer cell, tissue explant, and animal models. Wound Repair Regen. 2007;15:94–104. 17244325 10.1111/j.1524-475X.2006.00190.x

[15] Lansdown ABS. I: Its antibacterial properties and mechanism of action. J Wound Care. 2002;11:125–30. 11998592 10.12968/jowc.2002.11.4.26389

[16] Lansdown AB. Silver. 2: toxicity in mammals and how its products aid wound repair. J Wound Care. 2002;11:173–7. 12055941 10.12968/jowc.2002.11.5.26398

[17] Mooney EK Lippitt C Friedman J. Silver dressings. Plast Reconstr Surg. 2006;117:666–9. 16462356 10.1097/01.prs.0000200786.14017.3a

[18] Poon VK Burd A. In vitro cytotoxity of silver: implication for clinical wound care. Burns. 2004;30:140–7. 15019121 10.1016/j.burns.2003.09.030

[19] Barnea Y Amir A Leshem D. Clinical comparative study of aquacel and paraffin gauze dressing for split-skin donor site treatment. Ann Plast Surg. 2004;53:132–6. 15269581 10.1097/01.sap.0000112349.42549.b3

[20] Chaby G Senet P Vaneau M. Dressings for acute and chronic wounds: a systematic review. Arch Dermatol. 2007;143:1297–304. 17938344 10.1001/archderm.143.10.1297

[21] Cohn SM Lopez PP Brown M. Open surgical wounds: how does Aquacel compare with wet-to-dry gauze? J Wound Care. 2004;13:10–2. 14969020 10.12968/jowc.2004.13.1.26556

[22] Guest JF Ruiz FJ. Modelling the cost implications of using carboxy- methylcellulose dressing compared with gauze in the management of surgical wounds healing by secondary intention in the US and UK. Curr Med Res Opin. 2005;21:281–90. 15801999 10.1185/030079905X25532

[23] Robinson BJ. The use of a hydrofibre dressing in wound management. J Wound Care. 2000;9:32–4. 10827666 10.12968/jowc.2000.9.1.25941

[24] Tachi M Hirabayashi S Yonehara Y. Comparison of bacteria-retaining ability of absorbent wound dressings. Int Wound J. 2004;1:177–81. 16722876 10.1111/j.1742-4801.2004.00058.x PMC7951750

[25] Williams C. An investigation of the benefits of aquacel hydrofibre wound dressing. Br J Nurs. 1999;8:676–7, 680. 10624199 10.12968/bjon.1999.8.10.6607

[26] Caruso DM Foster KN Blome-Eberwein SA. Randomized clinical study of hydrofiber dressing with silver or silver sulfadiazine in the management of partial-thickness burns. J Burn Care Res. 2006;27:298–309. 16679897 10.1097/01.BCR.0000216741.21433.66

[27] Coutts P Sibbald RG. The effect of a silver-containing hydrofiber dressing on superficial wound bed and bacterial balance of chronic wounds. Int Wound J. 2005;2:348–56. 16618321 10.1111/j.1742-4801.2005.00150.x PMC7951224

[28] Jude EB Apelqvist J Spraul M. Prospective randomized controlled study of hydrofiber dressing containing ionic silver or cal- cium alginate dressings in non-ischaemic diabetic foot ulcers. Diabet Med. 2007;24:280–8. 17305788 10.1111/j.1464-5491.2007.02079.x

[29] Jurczak F Dugre T Johnstone A. Randomised clinical trial of hydrofiber dressing with silver versus povidone-iodine gauze in the management of open surgical and trau-matic wounds. Int Wound J. 2007;4:66–76. 17425549 10.1111/j.1742-481X.2006.00276.x PMC7951761

[30] Kazmierski M Mankowski P Jankowski A. Comparison of the results of operative and conservative treatment of deep dermal partial-thickness scalds in children. Eur J Pediatr Surg. 2007;17:354–61. 17968794 10.1055/s-2006-924646

[31] Lohana P Potokar TS. Aquacel Ag in paediatric burns: a prospective audit. Ann Burns Fire Disasters. 2006;19:1–10. PMC3188096 21991040

[32] Mishra A Whitaker IS Potokar TS. The use of aquacel Ag in the treatment of partial thickness burns: a national study. Burns. 2007;33:679–80. 17512668 10.1016/j.burns.2006.10.400

[33] Efron G Ger R. Use of surgical adhesive tape (steri-strips) to secure skin graft on digits. Am J Surg. 1968;116:474. 4877582 10.1016/0002-9610(68)90249-3

[34] Harb A Pandya AN. Avoiding suture tie-over dressings on nasal dorsum. Plast Surg Int. 2010;2010:958213. 22567233 10.1155/2010/958213 PMC3335487

[35] Dewan P Smith A Ho M. Skin grafts: do we need to suture? J Am Acad Dermatol. 2009;11:554.

[36] Yen YH Lin CM Hsu H. Skin graft fixation using hydrofiber (aquacel extra). Ann Plast Surg. 2018;80:616–21. 29664827 10.1097/SAP.0000000000001432

